# Physical Activity Participation of Black and White Women during the First Year Postpartum: Results and Study Recruitment Strategies

**DOI:** 10.3390/healthcare11192625

**Published:** 2023-09-26

**Authors:** Erin E. Kishman, Lauren A. Castleberry, Xuewen Wang

**Affiliations:** 1 Department of Exercise Science, School of Public Health, University of South Carolina, 921 Assembly St., PHRC 301, Columbia, SC 29208, USA; 2 Department of Obstetrics and Gynecology, Lexington Medical Center, West Columbia, SC 29169, USA

**Keywords:** postpartum, physical activity, sedentary time, recruitment, retention

## Abstract

**Background**: Little is known about how physical activity participation changes throughout the first year postpartum. This may be due to the difficulty in recruiting this population. The aims of this study were first to describe the recruitment methods and second to examine physical activity in the first year postpartum. **Methods**: Black and White women who gave birth to a singleton infant at ≥37 weeks gestation were recruited by a variety of strategies. At 6–8 weeks (baseline) and 4, 6, 9, and 12 months postpartum, women were instructed to wear an accelerometer for 7 days. **Results**: Active recruitment with interactions between staff and potential participants was more successful than non-active strategies for enrolling women. Throughout the first year postpartum, physical activity counts and light and moderate-to-vigorous physical activity increased from baseline (165.2 and 21.5 min, respectively) to 12 months (185.0 and 27.6 min, respectively). Sedentary time decreased from 775.3 min at baseline to 749.4 min per day at 12 months. In this sample, Black participants had lower physical activity (counts per minute per day) and greater sedentary time than White participants. **Conclusions**: Active strategies were more successful in recruiting women into the study. Of those who enrolled, physical activity levels increased over time. Identifying barriers to physical activity that may change over the postpartum period will help develop more targeted interventions to increase physical activity.

## 1. Introduction

Physical activity provides many health benefits. Higher levels of aerobic physical activity have been associated with decreased risks for mortality, cardiovascular diseases, metabolic syndrome, obesity, and improved mental health [1,2,3,4,5]. In the postpartum period, more physical activity has been associated with less risk for postpartum weight retention, decreased risk for postpartum depression, and better overall maternal well-being [6,7,8,9]. Physical activity guidelines for postpartum women are similar to those for the general adult population, primarily consisting of 150 min of moderate-to-vigorous aerobic physical activity (MVPA) a week [10]. Time from delivery is not specified in the guidelines; however, a woman’s participation in physical activity is likely to change in the postpartum period since recovering from delivery and caring for an infant can create new barriers to activity [11]. However, little is known about how physical activity participation changes in the postpartum period. 

Studies that have examined MVPA in the postpartum period show highly variable results. One study found no change in MVPA from 3 months to 12 months postpartum [12], and another study did not find significant differences in MVPA at 3, 6, 9, and 12 months postpartum [13]. However, other studies found a small increase in MVPA from 6.9 to 8.8 min per day over a 10-month period beginning 2–7 months postpartum [14] and an increase from 55.5 to 64.5 min per day in the first two months postpartum [15]. 

There is also evidence that sedentary time and time spent in light-intensity physical activity (LPA) are associated with health outcomes [16,17], making them important to include when examining physical activity levels. A few studies have found sedentary time to decrease and LPA to increase during postpartum [12,13,15]. However, the studies differ in when the measures were taken after delivery and the criteria used to determine the intensity of physical activity, which makes it difficult to compare across studies. 

Additionally, in the general adult population, Black adults are reported to have lower levels of physical activity when compared to White adults [18], which may contribute to health disparities. However, none of the abovementioned studies examining physical activity in the postpartum period have examined potential racial differences. Race captures structural, social, and cultural factors that are experienced differently between groups [19]. A reason for the lack of studies on postpartum women may be due to difficulties in recruiting women during this period [20,21,22,23]. Previous studies have found a lack of time due to childcare and returning to work as barriers for postpartum women to enroll in studies [20,21]. Some populations may experience additional barriers to enrollment, such as a lack of trust or knowledge about clinical research [22,23]. Thus, sharing recruitment strategies can help researchers plan their future recruitment protocol to adequately power their studies and improve the generalizability of the results. 

Therefore, the purpose of this paper is twofold. The first is to describe the physical activity and sedentary time during the first year postpartum in a sample of Black and White women. The second is to report the recruitment strategies, yield, and retention of Black and White postpartum women from a recently completed prospective cohort study. 

## 2. Methods

Data are from a longitudinal cohort study, the Postpartum Weight and Sleep (PWAS) Study, in women, followed from 6 to 8 weeks to 12 months postpartum [24]. In the study, enrolling 120–134 women was planned in order to retain 96 women (50% Black and 50% White), which would allow examining whether race significantly interacts with time on the outcome measures with adequate power. Participants were recruited from the Midlands area of South Carolina from October 2018 through January 2020. Study visits were between October 2018 and November 2020, during which the COVID-19 pandemic occurred. The main recruitment location, Columbia, SC, has a population of 52.6% White and 39.6% Black [25]. The study was conducted according to the guidelines of the Declaration of Helsinki and approved by the University of South Carolina Institutional Review Board (Protocol number: Pro00076434, approved on 25 April 2018). Informed consent was obtained from all participants involved in the study.

The inclusion criteria were women ≥18 years of age who delivered a singleton at ≥37 weeks gestation, self-identified as Black or White, and were providing care for the infant. Women were excluded if they self-reported diseases or medications, such as thyroid disease, diabetes, sleep apnea, clinical depression, or the use of the contraceptive Depo-Provera.

### 2.1. Recruitment Process

The first visit for participants in the study occurred between 6 and 8 weeks postpartum. Therefore, recruitment targeted women who were late into their pregnancy or had recently given birth. An interest form was developed with the intention for potential participants to sign up for the study before delivery and soon after delivery. This form asked women to identify their race, indicate if they plan to provide care for their baby, and if they plan on using the contraceptive Depo-Provera during postpartum. The form was also used to collect their contact information, delivery or due date, and their preferred time to be contacted. If deemed eligible from the interest form, potential participants were called to complete the full screening process. The full screening included questions about race, age, gestation duration, delivery date, if they were providing care for the infant, if they currently used contraceptives, and if they had any medical conditions. If a woman was eligible, she was scheduled for the baseline visit between 6 and 8 weeks after the delivery date. Participants were enrolled in the study once the baseline visit was completed. An email was used to complete the full screening if they could not be reached by phone. Women were contacted up to 5 times to conduct the full screening. If an interested individual reached out to us, we skipped the interest form and completed the full screening as appropriate. 

Participants were recruited from several sources, including obstetric clinics, labor and delivery units, a lactation and newborn clinic, social media, radio stations, and local events. Flyers, including brief information about the study, were designed to be used and distributed at these resources. At the obstetric clinics, research staff approached women in the waiting room before their appointment with healthcare providers or centering pregnancy meetings. Physicians informed their patients of the study during their appointment. Interest forms and flyers were also placed in the waiting rooms of obstetric clinics. The lactation and newborn clinic included the interest form in the paperwork women received before their visit. In the labor and delivery units, research staff approached women in their rooms. At local events, such as community baby showers and mom expos, research staff set up a booth and provided information about the study. Advertisements for the study were broadcasted on a radio station and placed on social media and blogs aimed towards mothers and women in the surrounding area. The blogs included a post about the study on their website, which was also posted on their social media accounts. Flyers of the study were distributed around the community, including a pediatric clinic, a daycare, university buildings, and shops for maternity and infant clothing. Participants who contacted us but did not indicate where and how they learned about the study were defined as self-referred.

In order to recruit and retain participants throughout the study, we attempted to reduce the participant’s burden as much as possible. A flexible schedule was maintained to allow participants to come when it was convenient. Before visits, research staff called or emailed the participants to remind them of their upcoming visit. During the visit, childcare was provided. To compensate the participants for their time, a USD 50 incentive was given at each time point once the participants completed the procedures. 

### 2.2. Physical Activity Measurement 

Physical activity was measured via actigraphy (GT3X+; Actigraph, Pensacola, FL, USA) at 6–8 weeks, 4 months, 6 months, 9 months, and 12 months postpartum. At each time point, the participants were instructed to wear the monitor on their hip for 7 days and to only remove the monitor for sleeping and water activities. Salt water can damage the devices, and the hip belt to hold the device in place was not waterproof. Participants kept a log of when the monitor was removed and put back on. Data were collected at a sampling rate of 30 Hz in 10 s epochs and were analyzed using the manufacturer-provided software (ActiLife software version 6.13.4, Pensacola, FL, USA). The Choi algorithm was used to determine the non-wear time, which defines 90 consecutive minutes of zero counts as non-wear [26]. A valid day of wear time was defined as 600 or more minutes of wear [27]. Participants with at least three valid days of wear time were included in the physical activity analysis according to the recommendations [28]. Counts per minute (CPM) were used to determine the time spent sedentary and doing LPA, moderate PA (MPA), and vigorous PA (VPA). Two sets of cut points, Swartz et al. [29] and Troiano et al. [27], which have previously been used in postpartum women, were applied. Sedentary behavior was defined as <100 CPM for both. Troiano et al. [27] defines LPA as 100–2019 CPM, MPA as 2020–5998 CPM, and VPA as ≥5999 CPM. Swartz et al. [29] defines LPA as 100–573 CPM, MPA as 574–4944 CPM, and VPA as ≥4945.

### 2.3. Statistical Analysis

Descriptive statistics for the participant characteristics were calculated. To examine the differences at baseline between the enrolled and dropped participants and to examine the differences by race, t-tests or chi-square tests were used, as appropriate. Mixed-effects linear models were used to examine the changes in sedentary time, CPM, LPA, and MVPA per day. The initial models included race and the race-by-time interaction to examine whether the changes over time were not different between the two races. In follow-up analyses, differences between time points were adjusted using the Bonferroni post hoc test for multiple comparisons. The covariates examined included age, income, education, employment, body mass index (BMI) status (normal, overweight, obese), parity, breastfeeding status (exclusively, some, none), and mode of delivery (vaginal or cesarean). If a moderate association (*p* < 0.10 for Pearson or Spearman’s rank correlation) with the outcome variable was found, the covariates were included in the model. The final model was adjusted for actigraph monitor wear time, age, income, education, employment, BMI status, and parity. The statistical significance was defined as a *p*-value < 0.05. Analyses were performed using SAS 9.4 (SAS Institute, Cary, NC, USA).

## 3. Results 

### 3.1. Recruitment Yield and Recruitment Strategy

**Figure 1:**
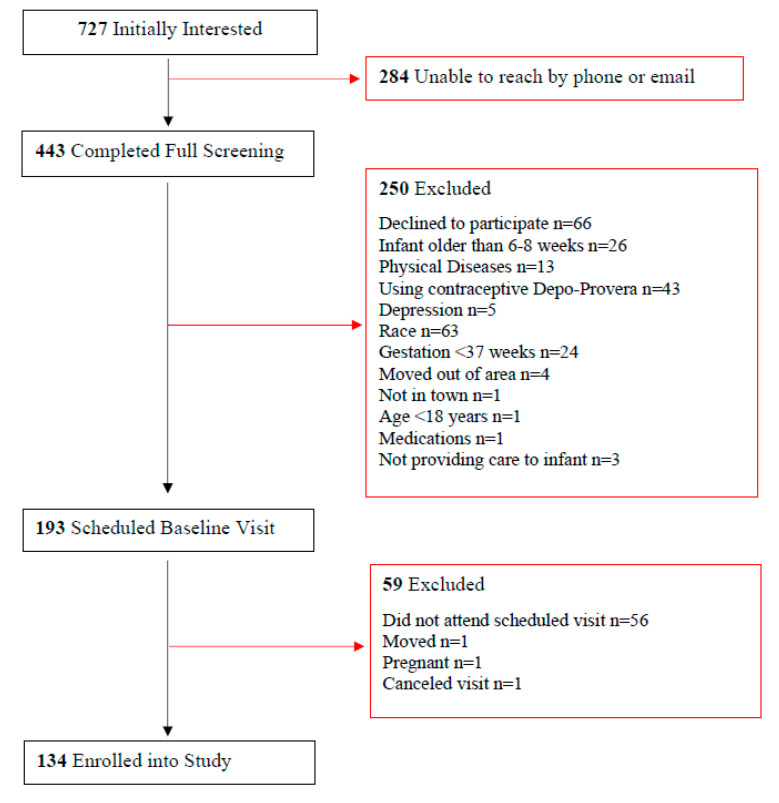
Participant enrollment flow chart.

The recruitment process and exclusion reasons are displayed in Figure 1. Of the 727 initially interested women, 39% were unable to be reached by phone or email. During screening, the most common reasons for exclusion were declining to participate (26%), not self-identifying as Black or White (25%), and using the contraceptive Depo-Provera (17%). After scheduling the first visit, 59 women were excluded. The main reason for exclusion was not showing up to their scheduled visit (95%). A total of 134 women, 18.4% of all women initially interested, enrolled in the study.

**Figure 2:**
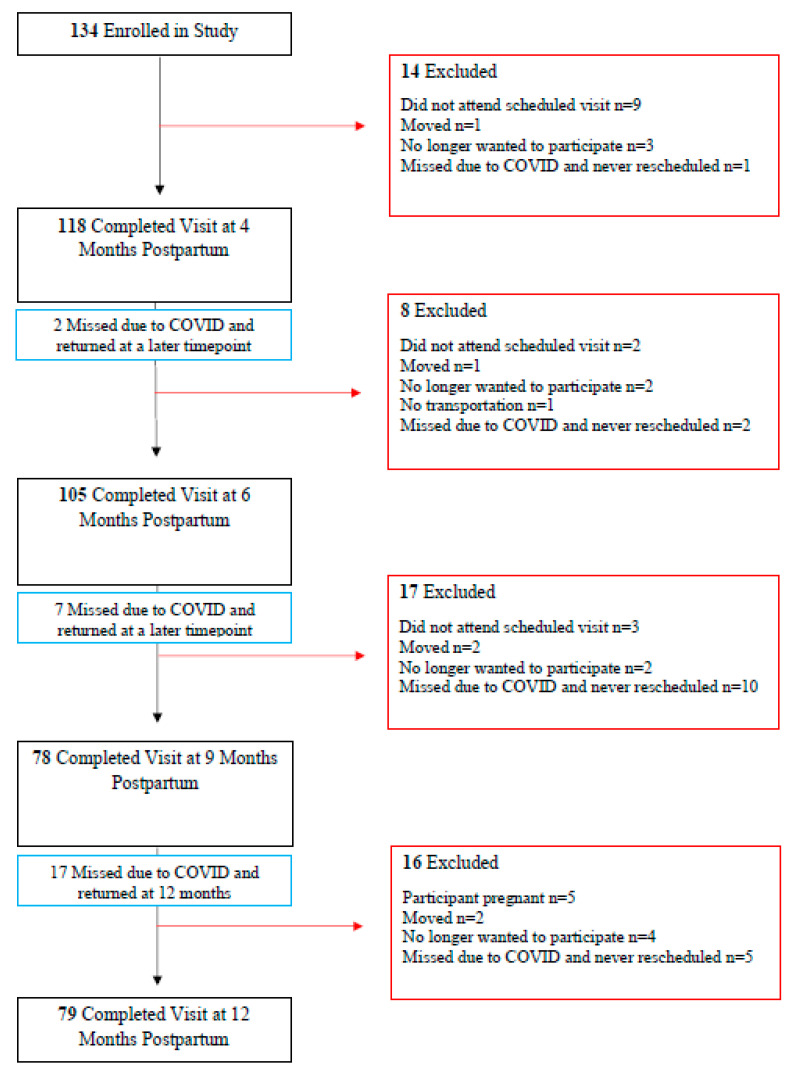
Participant retention flow chart.

The retention at each time point is displayed in Figure 2. Of the 134 women who enrolled in the study, 55 dropped at various points, and 79 completed the 12-month visit. This study was impacted by closures due to the COVID-19 pandemic. The University of South Carolina closed in March 2020, and we were unable to commence the participant visits again until June 2020. There were 26 missed visits during the closure where the participant remained in the study and continued visits once the campus was open again. The main reason for dropping out of the study was loss of follow-up. Fourteen participants (25%) did not attend their scheduled visits, and eighteen participants (33%) did not respond when we contacted them to reschedule their visits that were missed due to campus closure. 

Table 1 displays the recruitment yield by strategy for those who were interested, enrolled, and completed the study. The more successful strategies were active strategies, where research staff, physicians, or clinic staff were handing out the interest forms or discussing the study with potential participants. Active strategies (labor and delivery units, direct contact with research staff or physicians at obstetric clinics, lactation and newborn clinic, and community events) accounted for 87% of those interested in the study and 75% of those who enrolled in the study. 

Strategies that were successful in recruiting participants also had high levels of retention (Table 1). The lactation and newborn clinic was the most successful in recruiting both Black and White women to enroll in the study and had a retention rate (% of enrolled) of 64%. Blogs, self-referral, and physician recruitment at clinics had retention rates near or above 50%. A few strategies only enrolled one or two participants (research staff at obstetric clinics, interest forms at obstetric clinics, community events, and flyers) but these participants completed the study and, therefore, had a high retention rate. 

### 3.2. Characteristics of Enrolled Participants

The baseline participant characteristics are displayed in Table 2. Of the 134 participants who enrolled in the study, 63% were White, and 37% were Black. The age ranged from 18 to 43 years, with a mean age of 30 years. Regarding BMI, 39% were obese, and 38% were overweight. The majority of participants had graduated college or above (59%), were in a stable relationship (88%), and were employed (68%). Half of the participants had a household income greater than USD 60,000. For 39% of the participants, this was their first live birth. The majority had a vaginal delivery (73%). When examining the characteristics by race, Black participants were younger, had a larger proportion being obese, were less likely to be married or in a stable relationship, had a lower proportion of breastfeeding exclusively, and had a lower proportion in higher education and higher family income categories compared to White participants. There was no difference in the type of delivery, parity, or employment between races. 

The comparisons of the characteristics of participants who completed the 12-month visit and those who initially enrolled but dropped out without completing the study are in Table 3. Those who completed the study compared to those who dropped from the study were, on average, 2.3 years older, included more White women, had a higher proportion of breastfeeding, had higher education levels, and had a higher percentage of meeting the physical activity guidelines. The mean weight, relationship status, family income, and employment were not different between the women who completed and who dropped out of the study. 

### 3.3. Physical Activity 

The participants wore the actigraph for an average of 6 days (range 3–10 days) with an average wear time of 965 ± 190 min per day. The sample size of those with valid data at each time point was 130 (48 Black and 82 White) at 6–8 weeks, 109 (33 Black and 76 White) at 4 months, 99 (26 Black and 73 White) at 6 months, 75 (17 Black and 58 White) at 9 months, and 77 (18 Black and 59 White) at 12 months. 

The initial mixed-effects models did not find a significant race-by-time interaction, suggesting the changes in CPM per day, and sedentary, MVPA, and LPA time were similar between the Black and White participants. However, Black participants had a lower CPM per day (Pooled mean (standard error) of five time points: 377.7 (35.1) for Black, and 464.0 (29.0) for White, *p* < 0.001) and a greater sedentary time (781.6 (13.8) minutes for Black, and 759.1 (11.4) minutes for White, *p* = 0.026) than White participants. Black participants spent less time in MVPA than White participants when using the Swartz et al. [29] cut points (*p* = 0.033) but did not differ when the Troiano et al. [27] cut points were used (*p* = 0.058). The LPA time was not different between Black and White participants.

**Figure 3:**
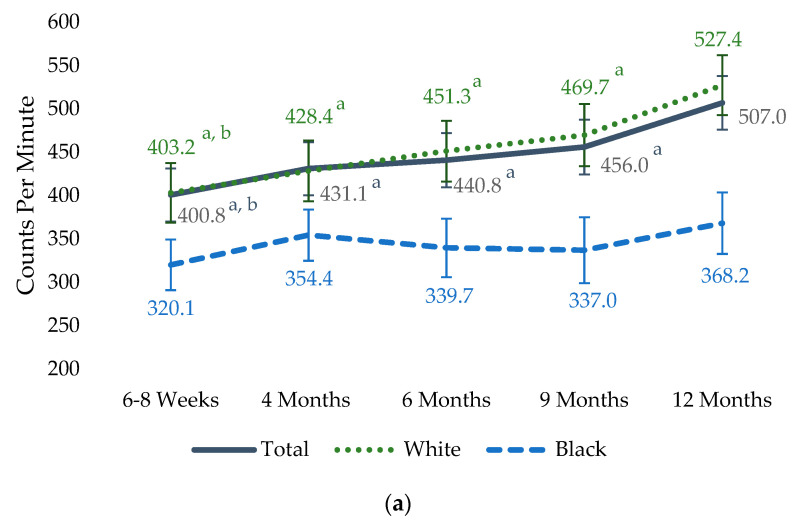

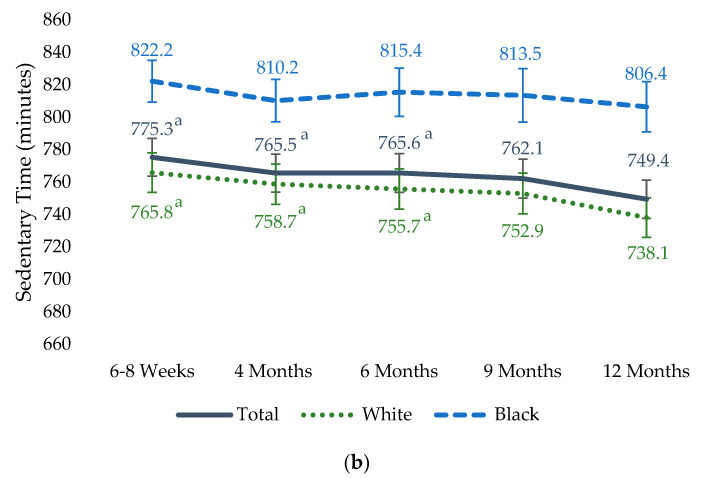
Data are least-square means, and the error bars are standard errors from mixed-effects models for (**a**) counts per minute and (**b**) sedentary time per day. Wear time, income, education, employment, BMI status, parity, and age were adjusted for in the models. Sample size: 48, 33, 26, 17, and 18 Black, and 82, 73, 58, and 59 White participants at 6–8 weeks, 4 months, 6 months, 9 months, and 12 months, respectively. ^a^ indicates Bonferroni adjusted *p* < 0.01 when compared to 12 months. ^b^ indicates Bonferroni adjusted *p* < 0.01 when compared to 9 months.

Subsequently, mixed-effects models were conducted in the entire sample and by race to examine changes over time. Figure 3 displays the adjusted means and standard errors from these models for the CPM and sedentary time per day at each time point. In the entire sample, physical activity counts (CPM per day) were lower at 6–8 weeks, 4 months, 6 months, and 9 months compared to 12 months postpartum (Bonferroni adjusted *p*-value ranged from <0.001 to 0.009). The CPM was also lower at 6–8 weeks compared to 9 months postpartum (Bonferroni adjusted *p* = 0.009). Sedentary time was higher at 6–8 weeks, 4 months, and 6 months when compared to 12 months postpartum (Bonferroni adjusted *p* < 0.001, 0.007, and 0.009, respectively). In White participants, the trends of change across the five time points for the CPM and sedentary time were similar to those of the entire sample. However, with Black participants, there were no statistically significant changes in the CPM or sedentary time in the first year postpartum.

Table 4 displays the results from the mixed-effects models for the LPA and MVPA time by using the Troiano et al. [27] and Swartz et al. [29] cut points. The amount of time in LPA and MVPA varied depending on the cut points used, as expected. When using the Troiano et al. [27] cut points, the LPA time was greater and the MVPA time was less by approximately 70 min than when using the Swartz et al. [29] cut points. In the entire sample, the LPA time at 6–8 weeks was less than at 12 months postpartum (Bonferroni adjusted *p* values < 0.001), and the MVPA time was less at 6–8 weeks, 4 months, and 6 months compared to 12 months postpartum (Bonferroni adjusted *p* values ≤ 0.011), regardless of the cut points used. 

In our White participants, the MVPA time was less at 4 and 6 months than 12 months postpartum using either cut points (the Bonferroni adjusted *p*-value ranged from <0.001 to 0.028). The MVPA at 6–8 weeks was also less than at 12 months using the Swartz et al. [29] cut points (Bonferroni adjusted *p* = 0.011). The LPA time was less at 6–8 weeks and 4 months compared to 12 months when using either cut points (the Bonferroni adjusted *p*-value ranged from <0.001 to 0.021), and the LPA at 6 months was less than at 12 months using the Troiano et al. [27] cut points (Bonferroni adjusted *p* = 0.036). In our Black participants, there were no statistically significant changes in the LPA or MVPA when using either cut points. 

To determine if the participants were meeting the physical activity guidelines, the mean daily MVPA was used to estimate the weekly MVPA. Analyses included all participants with valid data (at least 3 days with 600 min or more wear time each day) at each time point. When using the Swartz et al. [29] cut points, 100% of the participants met the physical activity guidelines at each time point. Using the Troiano et al. [27] cut points, the percentage of Black participants meeting the guidelines was 33%, 55%, 54%, 58%, and 78%, respectively. The percentage of White participants meeting the guidelines was 62%, 62%, 64%, 74%, and 76%, respectively. The percentage of White participants meeting the guidelines at 6–8 weeks was significantly higher than the Black participants (*p* = 0.0015). At the other time points, there were no statistically significant differences between the percentage of Black or White participants meeting the guidelines. 

## 4. Discussion

Recruitment of this population was difficult, and the enrollment yield was 18.4%. Active recruitment strategies appeared to be more successful. Over the first year postpartum, participation in physical activity increased, and sedentary time decreased in the entire sample and in White participants. No significant changes over time were found in Black participants, which may be due to the small sample size at later time points. Also, Black participants had more sedentary time and had a smaller amount of total physical activity (CPM per day) than White participants, with an adjustment for the covariates.

When examining the recruitment and retention rates by strategy, our active recruitment strategies were more successful than non-active ones. The success of active recruiting has been seen in other studies as well [30,31,32]. For recruiting both Black and White women, the lactation and newborn clinic was one of the successful strategies. Each woman who entered the clinic was given an interest form from clinic staff, which may have led to the high interest and retention of this strategy. Recruiting in the labor and delivery units was another one of our successful strategies, even though we started 6 months later than several other strategies. Additionally, when we started this strategy, we were almost at our recruitment goal for White women and thus stopped recruiting White women while we continued recruiting Black women, explaining why the number of White women recruited was lower at this site. One successful non-active strategy in our study was recruiting through blogs aimed towards mothers. This strategy also had one of the better retention rates. It was more successful in recruiting White than Black women; other blogs that Black women frequently visit may be more successful at recruiting Black women.

Few studies have objectively examined physical activity in the postpartum period. Since there are no cut points specifically for postpartum women to determine the intensity of activity, our data were analyzed using the cut points previously validated in adults that have been used in this population [27,29]. The cut points used made a large difference in the amount of MVPA and LPA time accumulated. When using the Troiano et al. [27] cut points, more time was spent in LPA and less time was spent in MVPA when compared to using the Swartz et al. [29] cut points. When examining the percentage of participants meeting the physical activity guidelines, based on the Swartz et al. [29] cut points, 100% of the participants were meeting the guidelines. It is unlikely that 100% of the participants were meeting the activity guidelines, and therefore, the Swartz et al. [29] cut points may be overestimating MVPA in postpartum women. Thus, validation of cut points used, specifically for the postpartum population, is recommended. We will focus on discussing our results from using the Troiano et al. [27] cut points. 

Although MVPA and LPA were not significantly different between our Black and White participants, Black participants had more sedentary time and lower total physical activity (CPM per day) than White participants. Similar results in the general adult population have been reported previously [18]. Our study extended these findings to women in the postpartum period. The percentage of participants meeting the guidelines, i.e., 150 min of MVPA a week, was lower in Black women compared to White women at 6–8 weeks postpartum; however, from 4 to 12 months postpartum, they were not significantly different. This could partially be due to the characteristics of physical activity participation in those who dropped out. Black women who dropped from the study after the 6–8 weeks’ visit had a lower proportion of meeting the guidelines compared to those who continued to complete the month 4 visit (25% versus 36%). Also, Black women in our study may experience different barriers to physical activity in the early postpartum weeks than later postpartum. A previous study found fatigue and time constraints due to caring for the infant as possible barriers to physical activity [11]. Identifying barriers that may change over time will help develop more targeted interventions.

When examining the longitudinal changes in MVPA, there were significant increases of about 5–6 min per day on average in the overall sample and in White participants, from 6 to 8 weeks, 4 months, and 6 months to 12 months. No significant changes were found in the Black participants in our study; however, our sample size of Black women became small after 4 months postpartum; thus, the non-significant change over time may be due to the sample size. However, in a previous study with predominately White women, Evenson et al. [12] did not find significant changes in MVPA from 3 months (17 min per day) to 12 months (18 min per day) postpartum when using the Troiano et al. [27] cut points. In another study with primarily Black women, Hesketh et al. [13] also did not find significant changes in MVPA from 3 months (10.0 min per day) to 12 months (12.8 min per day) postpartum when using the Troiano et al. [27] cut points. Compared to these two studies, the changes in MVPA in our study appear to be of bigger magnitude. Of note, our data also suggest the increase in MVPA appears to be more noticeable after 6 months postpartum. This information is valuable as we consider physical activity guidelines.

We also found a significant increase of about 20 min in time spent in LPA from 6 to 8 weeks to 12 months postpartum in the overall sample and in White participants. In our Black participants, there were no statistically significant changes in LPA; this may result from a smaller increase and could also be due to the smaller sample sizes at later visits. Also, our data suggest a gradual increase in LPA over the period from early to 12 months postpartum rather than a more noticeable change at a certain time point. The previous study with primarily Black participants found an increase in LPA from 259.6 min at 3 months to 296.9 min at 12 months postpartum [13], while the study of primarily White participants found no change in LPA from 3 months to 12 months postpartum [12]. 

Although there are differences in our findings regarding the MVPA and LPA time changes compared to the two previous studies, which examined the longitudinal changes in the postpartum period, the trend of change in sedentary time was similar [12,13]. There were decreases in sedentary time and increases in the CPM per day throughout the first year postpartum in the entire sample and in White participants. Our Black participants had no significant changes in the CPM per day or sedentary time; this is likely due to a smaller magnitude of change and a small sample size completing the visits. Compared to the two previous studies [12,13], our Black and White participants spent slightly more time in MVPA, less time in LPA (by about 100 min per day), and much more time being sedentary. Their sedentary time ranged between 485 and 560 min, while our participants spent an average of about 750 to 775 min per day. A notable difference in the monitor wear time may explain some of these differences: the wear time in their studies was around 12 h a day, and our participants had an average of 16 h a day.

One of the main strengths of this study is that it objectively measured physical activity in a sample of Black and White women at five time points throughout the first year postpartum, showing how physical activity changes during this period. We also reported on our recruitment strategies, which could benefit future studies to adequately plan their recruitment protocol. However, this study does have limitations. The sample who completed the study was older, had higher levels of education, and had higher amounts of physical activity than those who dropped out of the study, which may decrease the generalizability of the findings. The COVID-19 pandemic started while the study was underway. The pandemic may affect women’s physical activity levels, and we were not able to measure the impact. Additionally, our sample was Black and White women from South Carolina, United States, and thus, it may not be generalizable to other populations. Lastly, we had a smaller sample of Black women completing the study; caution should be used when interpreting the findings involving later time points with small sample sizes.

## 5. Conclusions

Recruitment and retention of participants were difficult in our study, with an 18.4% enrollment yield and a 41.0% attrition rate. Active recruitment strategies were more successful than non-active ones, emphasizing the importance of clinic or research staff interacting with women. Future studies are thus recommended to use active strategies to facilitate recruitment. Blogs could be successful if tailored to the specific population being recruited. This study also found that physical activity participation increased and sedentary time decreased throughout the first year postpartum. A lower percentage of women were meeting the guidelines in early postpartum; however, more were meeting the guidelines later in the first year postpartum. Black participants had lower physical activity participation and more sedentary time than White participants in our study. Further research is needed to understand the reasons, such as social, structural, and environmental factors, behind the differences between races in physical activity participation.

## Figures and Tables

**Table 1:** Recruitment yield by strategy, sorted by number of women initially interested in the study.

Recruitment Strategy	Initially Interested n (% of Total Interested)	Enrolled in Study n (% of Enrolled, % of Initially Interested in the Specific Strategy)			Completed Study n (% of Completed, % of Enrolled for the Specific Strategy)		
	Total n = 727	Total n = 134	White n = 85	Black n = 49	Total n = 79	White n = 58	Black n = 21
Lactation and Newborn Clinic	337 (46)	47 (35, 14)	25 (29, 7)	22 (45, 6)	30 (38, 64)	18 (31, 72)	12 (57, 55)
Labor and Delivery Units	183 (25)	30 (22, 16)	11 (13, 6)	19 (39, 10)	11 (14, 37)	5 (9, 45)	6 (28, 32)
Physician Recruitment at Obstetric Clinics	76 (10)	21 (16, 28)	15 (18, 20)	6 (12, 9)	10 (13, 48)	9 (15, 60)	1 (5, 17)
Blogs	36 (5)	20 (15, 56)	19 (22, 53)	1 (2, 3)	16 (20, 80)	15 (25, 79)	1 (5, 100)
Self-Referred	34 (7)	10 (7, 29)	9 (11, 26)	1 (2, 3)	8 (10, 80)	7 (12, 78)	1 (5, 100)
Research Staff at Obstetric Clinics	23 (3)	1 (1, 4)	1 (1, 4)	0 (0, 0)	1 (1, 100)	1 (2, 100)	0 (0, 0)
Community Events	21 (3)	1 (1, 5)	1 (1, 5)	0 (0, 0)	1 (1, 100)	1 (2, 100)	0 (0, 0)
Interest Forms at Obstetric Clinics	10 (1)	1 (1, 10)	1 (1, 10)	0 (0, 0)	1 (1, 100)	1 (2, 100)	0 (0, 0)
Flyers Placed at Various Locations	5 (<1)	2 (1, 40)	2 (2, 40)	0 (0, 0)	1 (1, 50)	1 (2, 50)	0 (0, 0)
Advertisements on Radio	2 (<1)	1 (1, 50)	1 (1, 50)	0 (0, 0)	0 (0, 0)	0 (0, 0)	0 (0, 0)

**Table 2:** Characteristics at 6–8 weeks postpartum of enrolled participants.

	Total (n = 134)	White (n = 85)	Black (n = 49)	*p*-Value
**Age (years)**	30.4 ± 4.8	31.7 ± 4.1	28.0 ± 5.1	<0.0001
**Weight (kg)**	79.7 ± 18.5	76.9 ± 15.1	84.6 ± 22.6	0.002
**BMI Status**				0.036
Normal	31 (23)	22 (26)	9 (18)	
Overweight	51 (38)	37 (44)	14 (29)	
Obese	52 (39)	26 (30)	26 (53)	
**Parity**				0.715
Primiparous	50 (39)	33 (40)	17 (37)	
Multiparous	78 (61)	49 (60)	29 (63)	
**Breastfeeding Status**				0.002
Exclusively	93 (69)	68 (80)	25 (51)	
Some	15 (12)	8 (9)	7 (15)	
None	26 (19)	9 (11)	17 (34)	
**Type of Delivery**				0.461
Vaginal	95 (73)	61 (75)	34 (69)	
Cesarean section	35 (27)	20 (25)	15 (31)	
**Relationship Status**				0.001
Married or stable relationship	118 (88)	82 (97)	36 (74)	
Married or stable relationship but not living with partner	7 (5)	1 (1)	6 (12)	
Divorced or widowed	3 (2)	2 (2)	1 (2)	
Not in a relationship	6 (5)	0 (0)	6 (12)	
**Education**				0.002
Some high school	1 (1)	1 (1)	0 (0)	
High school graduate	12 (9)	5 (6)	7 (14)	
College 1–3 years	42 (31)	18 (21)	24 (50)	
College graduate	40 (30)	31 (37)	9 (18)	
Graduate school	39 (29)	30 (35)	9 (18)	
**Income (USD)**				0.001
0 to 19,999	10 (8)	2 (2)	8 (16)	
20,000 to 39,999	30 (22)	17 (20)	13 (27)	
40,000 to 59,999	26 (19)	14 (17)	12 (25)	
60,000 to 79,999	20 (15)	11 (13)	9 (18)	
80,000 to 99,999	12 (9)	8 (9)	4 (8)	
100,000 to 120,000	16 (12)	15 (18)	1 (2)	
120,000+	20 (15)	18 (21)	2 (4)	
**Employment**				0.283
On maternity leave	57 (44)	40 (49)	17 (36)	
Employed	37 (29)	20 (24)	17 (36)	
Unemployed	35 (27)	22 (27)	13 (28)	

Data are means ± standard deviation or *n* (%). *p*-values are for comparison between Black and White women using a *t*-test or chi-square test. BMI: body mass index.

**Table 3:** Characteristics at 6–8 weeks postpartum of participants who completed the study versus participants who dropped from the study.

	Completed Study (n = 79)	Dropped (n = 55)	*p*-Value
**Age (years)**	31.3 ± 4.2	29.0 ± 5.4	0.009
**Weight (kg)**	80.4 ± 18.8	78.7 ± 18.2	0.612
**Percent Meeting Physical Activity Guidelines ^a^**	46 (58)	21 (38)	0.015
**Race**			0.004
White	58 (73)	27 (49)	
Black	21 (27)	28 (51)	
**Breastfeeding**			0.015
Exclusively	59 (75)	34 (62)	
Some	11 (14)	4 (7)	
None	9 (11)	17 (31)	
**Relationship Status**			
Married or stable relationship	71 (89)	47 (85)	0.397
Married or stable relationship but not living with partner	2 (3)	5 (9)	
Divorced or widowed	2 (3)	1 (2)	
Not in a relationship	4 (5)	2 (4)	
**Education**			0.001
Some high school	1 (1)	0 (0)	
High school graduate	2 (3)	10 (18)	
College 1–3 years	19 (24)	23 (42)	
College graduate	31 (39)	9 (16)	
Graduate school	26 (33)	13 (24)	
**Income (USD)**			0.217
0 to 19,999	3 (4)	7 (13)	
20,000 to 39,999	16 (20)	14 (25)	
40,000 to 59,999	17 (22)	9 (16)	
60,000 to 79,999	13 (16)	7 (13)	
80,000 to 99,999	5 (6)	7 (13)	
100,000 to 120,000	10 (13)	6 (11)	
120,000+	15 (19)	5 (9)	
**Employment**			0.063
On maternity leave	41 (52)	18 (33)	
Employed	18 (23)	21 (38)	
Unemployed	20 (25)	16 (29)	

Data are means ± standard deviation or *n* (%). *p*-values are for comparison between those who completed vs. those who dropped from the study using a *t*-test or chi-square test. ^a^: physical activity intensity determined using Troiano et al. [27] cut points.

**Table 4:** Light physical activity (LPA) and moderate-to-vigorous physical activity (MVPA) time (minutes) per day during the first year postpartum.

	Troiano			Swartz		
	Total	White	Black	Total	White	Black
LPA						
6–8 Weeks	165.2 (9.8) ^a^	163.5 (9.9) ^a^	150.0 (10.9)	95.3 (4.9) ^a^	96.4 (5.1) ^a^	85.0 (5.4)
4 Months	174.4 (9.8)	171.0 (10.1) ^a^	160.6 (11.0)	99.5 (4.9)	99.9 (5.2) ^b^	90.4 (5.5)
6 Months	174.4 (9.9)	173.1 (10.1) ^b^	156.8 (12.5)	100.4 (5.0)	101.1 (5.2)	91.2 (6.3)
9 Months	175.9 (10.0)	173.5 (10.3)	159.3 (13.9)	100.8 (5.0)	100.6 (5.3)	94.4 (7.0)
12 Months	185.0 (9.8)	185.3 (10.0)	160.0 (13.0)	104.6 (4.9)	106.5 (5.1)	89.9 (6.5)
MVPA						
6–8 Weeks	21.5 (3.1) ^a^	21.9 (3.9)	18.2 (3.1)	91.2 (7.9) ^a^	88.7 (8.5) ^a^	83.4 (8.6)
4 Months	22.1 (3.1) ^a^	21.5 (4.0) ^b^	19.6 (3.1)	97.1 (7.9) ^b^	93.0 (8.6) ^a^	89.6 (8.7)
6 Months	22.2 (3.2) ^a^	22.4 (4.0) ^b^	18.4 (3.6)	96.1 (8.0) ^a^	94.7 (8.6) ^b^	83.5 (9.9)
9 Months	24.1 (3.3)	24.7 (4.1)	18.0 (4.0)	99.2 (8.1)	97.9 (8.8)	82.2 (10.9)
12 Months	27.6 (3.2)	27.7 (3.9)	24.3 (3.7)	108.1 (7.9)	106.8 (8.5)	93.7 (10.3)

Data are least-square means and standard errors from mixed-effects models. Wear time, income, education, employment, BMI status, parity, and age were adjusted for in the models. Sample size: 48, 33, 26, 17, and 18 Black, and 82, 73, 58, and 59 White at 6–8 weeks, 4 months, 6 months, 9 months, and 12 months, respectively. ^a^ indicates Bonferroni adjusted *p* < 0.01 when compared to 12 months. ^b^ indicates Bonferroni adjusted *p* < 0.05 when compared to 12 months.

## Data Availability

Data sharing is available upon reasonable written request to the corresponding author and execution of a data sharing agreement.
